# Coherent full polarization control based on bound states in the continuum

**DOI:** 10.1038/s41467-022-31726-1

**Published:** 2022-08-04

**Authors:** Ming Kang, Ziying Zhang, Tong Wu, Xueqian Zhang, Quan Xu, Alex Krasnok, Jiaguang Han, Andrea Alù

**Affiliations:** 1 College of Physics and Materials Science, Tianjin Normal University, Tianjin, 300387 China; 2 Department of Electrical and Computer Engineering, The University of Texas at Austin, Austin, TX 78712 USA; 3 Center for Terahertz Waves and College of Precision Instrument and Optoelectronics Engineering, and the Key Laboratory of Optoelectronics Information and Technology (Ministry of Education), Tianjin University, Tianjin, 300072 China; 4 Photonics Initiative, Advanced Science Research Center, City University of New York, New York, NY 10031 USA; 5 Guangxi Key Laboratory of Optoelectronic Information Processing, School of Optoelectronic Engineering, Guilin University of Electronic Technology, Guilin, 541004 China; 6 Physics Program, Graduate Center, City University of New York, New York, NY 10016 USA

**Keywords:** Nanophotonics and plasmonics, Sub-wavelength optics

## Abstract

Bound states in the continuum (BICs) are resonant modes of open structures that do not suffer damping, despite being compatible with radiation in terms of their momentum. They have been raising significant attention for their intriguing topological features, and their opportunities in photonics to enhance light-matter interactions. In parallel, the coherent excitation of optical devices through the tailored interference of multiple beams has been explored as a way to enhance the degree of real-time control over their response. Here, we leverage the combination of these phenomena, and exploit the topological features of BICs in the presence of multiple input beams to enable full polarization control on the entire Poincaré sphere in a photonic crystal slab only supporting a symmetry-protected BIC, experimentally demonstrating highly efficient polarization conversion controlled in real time through the superposition of coherent excitations. Our findings open exciting opportunities for a variety of photonic and quantum optics applications, benefitting from extreme wave interactions and topological features around BICs combined with optical control through coherent interference of multiple excitations.

## Abstract (web summary)

Polarization control is of paramount importance for various applications. Here, the authors enable extreme control over light polarization spanning the entire Poincaré sphere by combining coherent control of wave phenomena and the physics of bound states in the continuum.

## Introduction

The electromagnetic field polarization is a manifestation of the vectorial nature of light, and its control is of paramount importance for various applications, including optical communications, quantum optics, sensing, and imaging. Polarization can be controlled through birefringent crystals, dichroic structures, optical gratings, Brewster phenomena, metamaterials, and complex materials^1–3^. Any arbitrary output polarization state can be obtained by properly selecting material anisotropy and input polarization. However, once the optical properties of a device and input polarization state are determined, the output polarization state is fixed and cannot be easily modified. Here, we explore the possibility of enabling highly flexible polarization control and tunability by combining coherent control of wave phenomena with the physics of optical embedded eigenstates (EEs)^4^.

An open resonator can support modes with zero radiation damping, known as EEs^4^ or bound states in the continuum (BICs)^5^, enabled by the destructive interference of two or more modes for a given frequency-momentum pair^6^. These states have been theoretically and experimentally demonstrated in various photonic platforms exploiting symmetry protection or accidental degeneracies^7–16^. Symmetry-protected BICs emerge if the bound state is in a different symmetry class compared to the radiation continuum, and hence radiation is not allowed as long as their symmetry is preserved. When the symmetry is lifted, a scattering line emerges, whose linewidth can be carefully controlled through the degree of broken symmetry, which tailors the ratio of stored energy and an overall loss in the system, sustained by absorption, radiation, and polarization conversion. Given that radiation damping is carefully controlled by symmetry breaking, the resonance linewidth can be carefully tuned and controlled, yielding remarkable features of great interest for various linear and nonlinear applications^17–23^.

Precise engineering of the different loss channels can result in various exotic phenomena, such as perfect reflection^24^, electromagnetically induced transparency (EIT)^25^, and exceptional point (EP) physics^26–29^. When the loss rates are matched to fulfill critical coupling, the resonator absorbs all incident light without scattering, enabling coherent perfect absorption (CPA)^30^. This phenomenon is enabled through the interference of multiple excitations, opening the possibility of widely controlling the system response in real time, going from unitary to zero absorption, as we change the relative phase of the excitation signals. In turn, this effect provides an efficient way to all-optical light control without requiring nonlinear phenomena^31–38^. CPA is not limited to Ohmic absorption, but it may be extended to other forms of energy conversion, including storage, polarization conversion, diffraction, and fluorescence^39–44^. CPA phenomena have been explored in various settings and offer applications for a broad range of light control and management^30,45^. Here we explore the real-time coherent control of nanophotonic devices supporting BICs.

A photonic crystal obeying ${C}_{4}$ rotational symmetry supports a symmetry-protected BIC at its Γ-point, associated with highly resonant guided modes emerging around this singularity and coupled to the far-field with very large *Q*-factors. A polarization vortex in momentum space with nontrivial topological features can be expected in the neighborhood of this singular scattering feature^5,46–51^. Complete polarization conversion (CPC) has been indeed theoretically explored encircling the BIC based on this response^46^. In such a configuration, the output polarization can be fully controlled by varying the incident angle, in order to effectively tune the radiative coupling rates of the two polarization channels around this CPC point, which coincides with a phase singularity in the excited polarization channel^52^.

In this work, we show that, by introducing coherent control by tuning the relative phase of multiple inputs, it is also possible to achieve *coherent complete polarization conversion* (CCPC), with polarization states spanning the entire Poincaré sphere. In our approach, the output polarization state can still be effectively tuned even at a fixed radiative coupling rate represented by a stationary incident angle, which adds a new degree of freedom for polarization manipulation. Our proposed coherent control of polarization conversion does not only dramatically expand the available polarization states for symmetry-protected BICs, but it also enables dynamic tuning with extreme flexibility. We experimentally demonstrate this flexible polarization control in the terahertz (THz) regime using a silicon photonic crystal slab exhibiting a symmetry-protected BIC, offering ideal features for THz polarimetric devices and overcoming challenges in efficiency and dynamic functionality^53^.

## Results

### Working principle

**Fig. 1:**
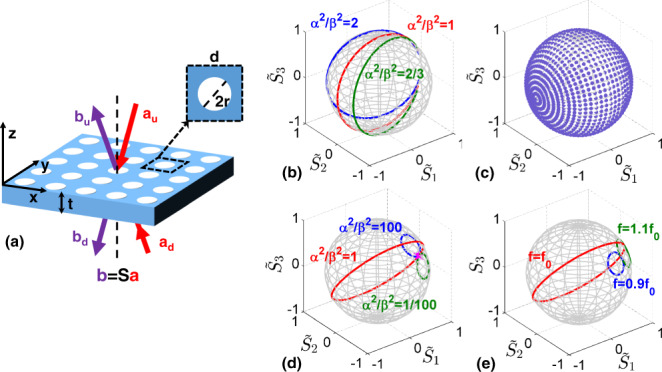
Polarization manipulation and its coherent control. **a** Schematic of the photonic crystal slab. **b**–**e** Polarization control as we vary: **b** the excitation frequency; **c** the background reflectivity and phase difference of the two input beams around the BIC point; **d** the phase difference of the two input beams at the BIC frequency with different radiative coupling rate ratio when operating at the BIC point $\alpha =\beta =0$ (scatter) and $\beta =0.01$,0.1,1 with fixed $\alpha =0.1$ (solid line); **e** the phase difference of the two input beams under the critical coupling condition $\alpha =\beta =0.1$. In **b** ${f}_{0}=1$, ${\alpha }^{2}=0.01$, ${\beta }^{2}=0.005,0.01,0.015$ and *f* varies from 0.9 to 1.1. In **d** ${r}_{0}=0.95$. In **e** ${r}_{0}=0.95$ and ${f}_{0}=1$. *N* = 0 is in all figures.

The optical features of resonant guided modes supported by a photonic crystal slab can be described using coupled-mode theory (CMT)^54^. As a starting point, we explore CCPC enabled by these resonant guided modes using CMT, in which cross-polarization coupling is treated as a loss channel. We consider a suspended slab with square lattice periodicity in both $x$ and $y$ directions and obeying mirror symmetry in $z$, as illustrated in Fig. 1a. The response of a guided resonance can be described in the steady-state regime by^54,55^

1 $$\begin{array}{l}[i\varDelta f+\gamma ]q={{{{{\bf{K}}}}}}{{{{{\bf{a}}}}}}\\ {{{{{\bf{b}}}}}}={{{{{\bf{C}}}}}}{{{{{\bf{a}}}}}}+{{{{{{\bf{K}}}}}}}^{{{{{{\rm{T}}}}}}}q\end{array},$$

where $\varDelta f=f-{f}_{0}$ is the detuning from the resonance frequency ${f}_{0}$, and $\gamma$ is the total decay rate, including all loss channels. We assume this resonant mode with a complex amplitude $q$ can only couple to the transverse-electric (TE) and transverse-magnetic (TM) zero-th order propagating plane waves without higher order diffraction. The input and output waves are $${{{{{\bf{a}}}}}}={(\begin{array}{cccc}{a}_{{{{{{\rm{u}}}}}}}^{{{{{{\rm{TE}}}}}}} & {a}_{{{{{{\rm{d}}}}}}}^{{{{{{\rm{TE}}}}}}} & {a}_{{{{{{\rm{u}}}}}}}^{{{{{{\rm{TM}}}}}}} & {a}_{{{{{{\rm{d}}}}}}}^{{{{{{\rm{TM}}}}}}}\end{array})}^{{{{{{\rm{T}}}}}}}$$ and $${{{{{\bf{b}}}}}}={(\begin{array}{cccc}{b}_{{{{{{\rm{u}}}}}}}^{{{{{{\rm{TE}}}}}}} & {b}_{{{{{{\rm{d}}}}}}}^{{{{{{\rm{TE}}}}}}} & {b}_{{{{{{\rm{u}}}}}}}^{{{{{{\rm{TM}}}}}}} & {b}_{{{{{{\rm{d}}}}}}}^{{{{{{\rm{TM}}}}}}}\end{array})}^{{{{{{\rm{T}}}}}}}$$, where the superscripts TE and TM correspond to the input and output polarizations, and the subscripts $u$ and $d$ denote the waves in the upper and lower semi-space with respect to the slab, respectively. ${{{{{\bf{C}}}}}}$ describes direct coupling usually modeled by scattering response of a homogeneous slab. The scattering matrix ${{{{{\bf{S}}}}}}$ around this resonance, defined by ${{{{{\bf{b}}}}}}={{{{{\bf{S}}}}}}{{{{{\bf{a}}}}}}$, can be expressed as

2 $${{{{{\bf{S}}}}}}={{{{{\bf{C}}}}}}+\frac{{{{{{{\bf{K}}}}}}}^{{{{{{\rm{T}}}}}}}{{{{{\bf{K}}}}}}}{i(\varDelta f+\gamma )}=\left(\begin{array}{cc}{{{{{{\bf{S}}}}}}}^{{{{{{\rm{TE}}}}}}} & {{{{{\bf{D}}}}}}\\ {{{{{\bf{D}}}}}} & {{{{{{\bf{S}}}}}}}^{{{{{{\rm{TM}}}}}}}\end{array}\right),$$

where ${{{{{{\bf{S}}}}}}}^{{{{{{\rm{TE}}}}}}}$ (${{{{{{\bf{S}}}}}}}^{{{{{{\rm{TM}}}}}}}$) indicates the scattering matrix of the resonant mode in the same polarization channel and ${{{{{\bf{D}}}}}}$ indicates the cross-polarized scattering matrix. The radiative coupling ${{{{{\bf{K}}}}}}$ between the resonant mode and the input waves is ${{{{{\bf{K}}}}}}=(\alpha {e}^{i({\zeta }^{{{{{{\rm{TE}}}}}}}+\sigma {\chi }^{{{{{{\rm{TE}}}}}}}+\pi )/2},\sigma \alpha {e}^{i({\zeta }^{{{{{{\rm{TE}}}}}}}\,+\,\sigma {\chi }^{{{{{{\rm{TE}}}}}}}+\pi )/2},\,\beta {e}^{i[{{{{{\rm{N}}}}}}\pi \,+\,({\zeta }^{{{{{{\rm{TM}}}}}}}+\sigma {\chi }^{{{{{{\rm{TM}}}}}}}+\pi )/2]},\,\sigma \beta {e}^{i[{{{{{\rm{N}}}}}}\pi \,+\,({\zeta }^{{{{{{\rm{TM}}}}}}}+\sigma {\chi }^{{{{{{\rm{TM}}}}}}}+\pi )/2]})$, where *α *(*β*) is the radiative coupling coefficient for TE (TM) polarized waves and N is an integer. $\sigma ={\pm}1$ indicates the resonant mode needs to be even or odd with respect to *z* due to the existent mirror symmetry. ${\zeta }^{{{{{{\rm{TE}}}}}},{{{{{\rm{TM}}}}}}}$ are global phase factors during direct coupling process and $\cos ({\chi }^{{{{{{\rm{TE}}}}}},{{{{{\rm{TM}}}}}}})$ indicate direct coupling reflection amplitudes. The total loss rate is $\gamma ={\alpha }^{2}+{\beta }^{2}+{\gamma }_{{{{{{\rm{d}}}}}}}$, where ${\gamma }_{{{{{{\rm{d}}}}}}}$ is the dissipation loss rate. The theoretical details can be found in Supplementary Note 1. Under a one port excitation, the loss in the original excited channel has an upper bound of 0.5 in a mirror-symmetric resonator^30^, and the detailed discussion is in Supplementary Note 2. This upper bound makes it impossible to entirely absorb light or totally convert polarization for a single input wave, due to symmetry, hindering applications relying on absorption or polarization conversion, including energy harvesting, sensing, and filtering^56,57^. In order to overcome this bound and maximize absorption or polarization conversion, mirror symmetry can be broken, but this requires a trade-off with the overall footprint and fabrication complexity. Excitation with multiple waves can also overcome this bound and realize full absorption or polarization conversion, as shown in the case of CPAs. Coherent control has indeed emerged as an attractive approach to enable unitary absorption in ultrathin resonant systems, also offering the opportunity for dynamic control by tailoring the relative phase between the inputs.

Without loss of generality, we consider the even mode of excitation of the system, $\sigma =1$. First, we explore the general condition for CPA in the case of multiple loss channels, including polarization conversion, which generalizes the conventional CPA condition limited to the presence of dissipation only. The symmetric eigenvector of ${{{{{{\bf{S}}}}}}}^{{{{{{\rm{TE}}}}}}}$ has a zero eigenvalue at a real frequency when

3 $$\varDelta f=0,\,{\alpha }^{2}={\beta }^{2}+{\gamma }_{{{{{{\rm{d}}}}}}}.$$

This condition corresponds to critical coupling^58^, for which the combination of polarization conversion to TM, described by $\beta$, and dissipation loss rate, described by ${\gamma }_{{{{{{\rm{d}}}}}}}$, contribute to the overall critical coupling to the input TE polarization. Similarly, the symmetric eigenvector of ${{{{{{\bf{S}}}}}}}^{{{{{{\rm{TM}}}}}}}$ has a zero eigenvalue when $\varDelta f=0$, ${\beta }^{2}={\alpha }^{2}+{\gamma }_{{{{{{\rm{d}}}}}}}$. If the resonant structure does not support polarization conversion, ${\beta }^{2}=0$ $({\alpha }^{2}=0)$, loss is limited to dissipation, and we achieve the usual CPA condition, which maximizes Ohmic absorption at $\varDelta f=0$, ${\alpha }^{2}={\gamma }_{{{{{{\rm{d}}}}}}}$ $({\beta }^{2}={\gamma }_{{{{{{\rm{d}}}}}}})$ for TE (TM) excitation from the two sides with the same amplitude and phase. Conversely, when the dissipation loss rate is zero ${\gamma }_{{{{{{\rm{d}}}}}}}=0$, polarization conversion is the only loss channel, giving rise to complete polarization conversion for $\varDelta f=0$ and ${\alpha }^{2}={\beta }^{2}$ under symmetric incidence.

The polarization state of the output waves can be generally described by the normalized Stokes parameters $({S}_{0} \;\; {S}_{1}/{S}_{0} \;\; {S}_{2}/{S}_{0} \;\; {S}_{3}/{S}_{0})$, which are controlled by the working frequency, scattering loss rates, and/or the relative phase difference of the input beams. We focus here on polarization control around the BIC, assuming absence of dissipation loss, i.e., ${\gamma }_{{{{{{\rm{d}}}}}}}=0$. In addition, we assume that the response of the background slab follows ${\chi }_{{{{{{\rm{TE}}}}}}}\approx {\chi }_{{{{{{\rm{TM}}}}}}}={\chi }_{0}$, ${\zeta }_{{{{{{\rm{TE}}}}}}}\approx {\zeta }_{{{{{{\rm{TM}}}}}}}={\zeta }_{0}$ close to the BIC, which implies nearly-normal incidence. The normalized Stokes parameters in the upper semi-space under symmetric TE incidence ${{{{{\bf{a}}}}}}={(1 \;\; 1 \;\; 0 \;\; 0)}^{{{{{{\rm{T}}}}}}}$ are ${S}_{{{{{{\rm{u}}}}}}0}=1$, ${\tilde{S}}_{{{{{{\rm{u}}}}}}1}={S}_{{{{{{\rm{u}}}}}}1}/{S}_{{{{{{\rm{u}}}}}}0}=1-8{\alpha }^{2}{\beta }^{2}/\varOmega$, ${\tilde{S}}_{{{{{{\rm{u}}}}}}2}={S}_{{{{{{\rm{u}}}}}}2}/{S}_{{{{{{\rm{u}}}}}}0}=4\alpha \beta ({\alpha }^{2}-{\beta }^{2})\cos [{{{{{\rm{N}}}}}}\pi ]/\varOmega$, ${\tilde{S}}_{{{{{{\rm{u}}}}}}3}={S}_{{{{{{\rm{u}}}}}}3}/{S}_{{{{{{\rm{u}}}}}}0}=4\alpha \beta \varDelta f\,\cos [{{{{{\rm{N}}}}}}\pi ]/\varOmega$, where $\varOmega =\varDelta {f}^{2}+{({\beta }^{2}+{\alpha }^{2})}^{2}$. The output intensity normalized by the incident intensity in each side is constant, ${S}_{{{{{{\rm{u}}}}}}0}=1$, which implies that the output power on each side is the same as the input power on each side without asymmetric output. At the resonance frequency $\varDelta f=0$, the output polarization state is linearly polarized with rotation angle ${{{{{\rm{atan}}}}}}[2\alpha \beta /({\alpha }^{2}-{\beta }^{2})]$, and perfect polarization conversion occurs at the critical coupling condition $\alpha =\beta$. At the BIC, with $\alpha =\beta =0$, the critical coupling condition becomes singular, which means that the output polarization remains the same as the incident polarization. By selecting a BIC mode with excepted radiative coupling profile, we enable to achieve efficient CCPC over a wide parameter range around this BIC. From the physical standpoint, this wide control and tunability is associated with the enhanced light–matter interactions and sensitivity around the BIC combined with the coherent control through multiple excitations.

As we deviate from the resonance frequency, ${\tilde{S}}_{{{{{{\rm{u}}}}}}2}=0$ at critical coupling, and the output polarization state continuously evolves along the unit circle in the ${\tilde{S}}_{{{{{{\rm{u}}}}}}1}$-${\tilde{S}}_{{{{{{\rm{u}}}}}}3}$ plane, touching the north pole ${\tilde{S}}_{{{{{{\rm{u}}}}}}3}=1$ for $\varDelta f=2{\alpha }^{2}\,\cos [{{{{{\rm{N}}}}}}\pi ]$ and the south pole ${\tilde{S}}_{{{{{{\rm{u}}}}}}3}=-1$ for $\varDelta f=-2{\alpha }^{2}\,\cos [N\pi ]$. As we consider both frequency and radiative coupling changes, the output polarization state can be flexibly controlled, as shown in Fig. 1b, even if we use a symmetric input polarization state, e.g., linearly polarized.

Besides polarization control through scanning frequency, the output polarization can also be controlled by the phase delay between the two input beams at a fixed frequency, showcasing the opportunities enabled by multiple coherent excitations, analogous to CPA. As an example, for TE incidence $${{{{{\bf{a}}}}}}={(\begin{array}{cccc}1 & {e}^{i\varPsi } & 0 & 0\end{array})}^{{{{{{\rm{T}}}}}}}$$, the output polarization state in the upper region at the resonant frequency $\varDelta f=0$ under critical coupling $\alpha =\beta$ around the BIC is

4 $$\left({\tilde{S}}_{{{{{{\rm{u}}}}}}1},\,{\tilde{S}}_{{{{{{\rm{u}}}}}}2},\,{\tilde{S}}_{{{{{{\rm{u}}}}}}3}\right)=(-{\cos} [\varPsi ],\,\sin [2{\chi }_{0}+{{{{{\rm{N}}}}}}\pi ]\,\sin [\varPsi ],\,-{\cos} [2{\chi }_{0}+{{{{{\rm{N}}}}}}\pi ]\sin [\varPsi ]).$$

The output intensity ${S}_{{{{{{\rm{u}}}}}}0}=1$ and the polarization state evolves over a unit circle with angle $2{\chi }_{0}$ around ${\tilde{S}}_{{{{{{\rm{u}}}}}}2}$ as we vary the relative phase of the input beams. The induced chirality of the output state is described by ${\tilde{S}}_{{{{{{\rm{u}}}}}}3}=-{\cos} [2{\chi }_{0}]\cos [N\pi ]\sin [\varPsi ]={(-1)}^{N}(1-2{|{r}_{0}|}^{2})\sin [\varPsi ]$, which can be efficiently controlled through both the background response and the phase delay. The maximum chirality difference is determined by the background response, $\delta {\tilde{S}}_{{{{{{\rm{u}}}}}}3}=2\,\cos [2{\chi }_{0}]=2(1-2{|{r}_{0}|}^{2})$. Also here, the output polarization state can span the entire Poincaré sphere even if the input polarization state is fixed to linear. As illustrated in Fig. 1c, the output polarization state can arbitrarily span the entire Poincaré sphere when $\varPsi$ is varied from 0 to $2\pi$ and ${\chi }_{0}$ spans from 0 to $\pi /2$.

Besides polarization manipulation at critical coupling empowered by coherent control, feasible polarization manipulation can also be achieved in a more general scenario, according to Eq. (2). As we know, the explored BIC exhibits a polarization vortex in the far-field^48,51,59^. Interestingly, the output polarization state is not affected by the phase delay when the slab is excited exactly at the BIC arising at the $\varGamma$ point, i.e., at the center of the polarization vortex, represented by the scatter point in Fig. 1d. However, the topological features around this singular point ensure a strong polarization transition as soon as we deviate from the BIC, consistent with the previous discussion. For different radiative coupling ratios at the BIC resonance, i.e., different positions of the polarization vortex, as illustrated in Fig. 1d, the critical coupling condition ensures wide polarization manipulation under coherent control. Combining radiative coupling variation around the BIC and phase delay between the two input beams, feasible polarization control can be also expected. When we deviate from the BIC, i.e., away from the polarization vortex, as shown in Fig. 1e, even at critical coupling the output polarization state is not significantly modified by the relative phase difference when we operate far from the BIC. Efficient polarization manipulation exploiting these tuning parameters, such as radiative coupling, frequency, and phase delay, is empowered by the CCPC, which coincides with a critical coupling condition. Therefore, CCPC is critical to feasible polarization control, which can be easily accessed in a wide range by choosing a BIC mode with desired radiative coupling profile.

### Experimental verification

**Fig. 2:**
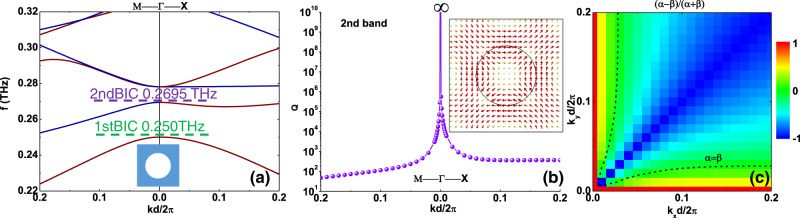
Symmetry-protected BIC in a photonic crystal slab. **a** Band structure of the silicon photonic crystal slab. The inset shows the schematic view of the unit cell of the designed photonic crystal slab. Red (blue) color denotes the guide mode that can efficiently couple to the incident TE (TM) polarization. **b** *Q*-factor of the 2nd band along the symmetry direction. The inset illustrates the eigenmode (**E** field) at *z* = 0. **c** Radiation coupling difference $(\alpha -\beta )/(\alpha +\beta )$ as a function of the wave vectors close to the 2nd BIC. The dashed line shows the critical coupling condition $\alpha =\beta$.

To experimentally verify efficient CCPC when operating around the BIC, we designed and fabricated a freestanding silicon photonic crystal slab supporting a symmetry-protected BIC in the THz region^5^. Figure 2 shows the band structure for *r* = 125.6 μm, *t* = 160 μm, and *d* = 475 μm, calculated using the guided-mode expansion (GME) method^60^. Two symmetry-protected BICs emerge at the Γ point below 0.32 THz, both with even symmetry $\sigma =1$. The *Q*-factor of the second mode is plotted in Fig. 2b, revealing the emergence of a BIC with diverging linewidth. As illustrated in the inset of Fig. 2b, the **E** field distribution at *z* = 0 within one unit cell reveals that the eigenmode is TE-like^48,51,59^. We choose to operate at the second BIC ~0.27 THz, because critical coupling for the leaky resonance can be more easily approached within the Brillouin zone near the BIC, as shown by the dashed line $(\alpha -\beta )/(\alpha +\beta )=0$ in Fig. 2c. In this panel, the condition $(\alpha -\beta )/(\alpha +\beta )=-1$ corresponds to nodal lines with *α* = 0, which indicates no radiative coupling with incident TE light along the $\varGamma {{{{{\rm{M}}}}}}$ direction, and nonzero radiative coupling with incident TM light $\beta \;\ne\; 0$. Conversely, when $(\alpha -\beta )/(\alpha +\beta )=1$ we find *β* = 0, which shows no radiative coupling with incident TM light along the $\varGamma {{{{{\rm{X}}}}}}$ direction, and nonzero radiative coupling with incident TE light $\alpha \;\ne\; 0$. At the $\varGamma$ point, intersection of these two nodal lines, both *α* = 0 and *β = 0*, implies the presence of a BIC. Around the BIC all these features can be accessed with small parameter variations, with the dashed lines in the figure indicating the critical coupling ensuring full polarization conversion.

**Fig. 3:**
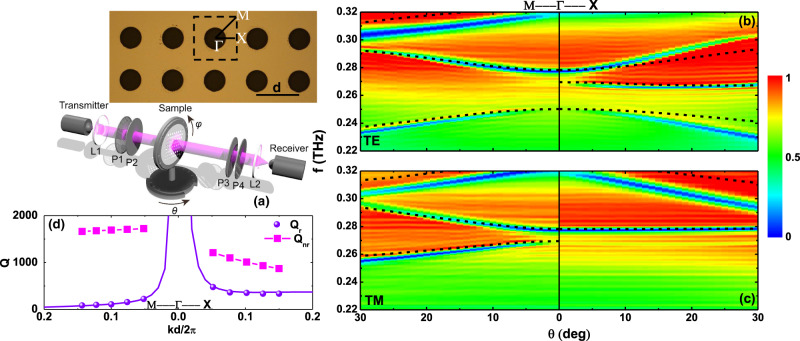
Angle-resolved transmission spectra identifying symmetry-protected BICs. Experimental setup (**a**) and measured transmission spectra along the symmetry directions for incident TE (**b**) and TM (**c**) polarized light. Dashed lines illustrate theoretical resonant guide modes for this slab. **d** Retrieved radiative (Scatter) and non-radiative (Dotted line) Q-factors along the two symmetry directions.

In order to confirm our theoretical predictions, a homemade fiber-based angle-resolved THz spectrometer was used to experimentally measure the transmission along two symmetric directions ($\varGamma {{{{{\rm{X}}}}}}$ and $\varGamma {{{{{\rm{M}}}}}}$ directions), as shown in Fig. 3a. The fabricated freestanding sample is 100*d* × 100*d*, as schematically shown in the inset of Fig. 3. The measured transmission spectra under TE and TM incidence clearly show a sharply resonant phenomenon, agreeing well with the theoretical prediction, as illustrated in Fig. 3b, c. Slight deviations between the measured results and numerical predictions may be due to fabrication imperfections. In the measured results we can observe a guided-mode (2nd mode) resonance stemming from a symmetry-protected BIC at 0.2695 THz, for which the resonant linewidth indeed vanishes when the incident angle approaches the Γ point. In particular, this mode shows different coupling along the two symmetric directions, as displayed in Fig. 3, in agreement with our prediction, and it can be used to match the critical coupling condition between the two symmetric directions. The radiative Q-factor ${Q}_{{{{{{\rm{r}}}}}}}$ retrieved from our measured transmission is well in agreement with our theoretical prediction, as shown in Fig. 3d, with details provided in Supplementary Note 3. As an example, in Supplementary Fig. 1, we provide fitting and measured transmitted intensities for $\theta ={15}^{{{{{{\rm{o}}}}}}}$ as a concrete example. The retrieved non-radiative Q-factor ${Q}_{{{{{{\rm{nr}}}}}}}$ due to dissipation loss from material absorption, scattering from imperfect fabrication, and finite excitation area of the slab, is large with respect to the radiative Q-factor in the measured incident angle range from $\theta ={10}^{{{{{{\rm{o}}}}}}}$ to $\theta ={30}^{{{{{{\rm{o}}}}}}}$, which implies that dissipation loss is small and does not play an important role in the proposed operation.

**Fig. 4:**
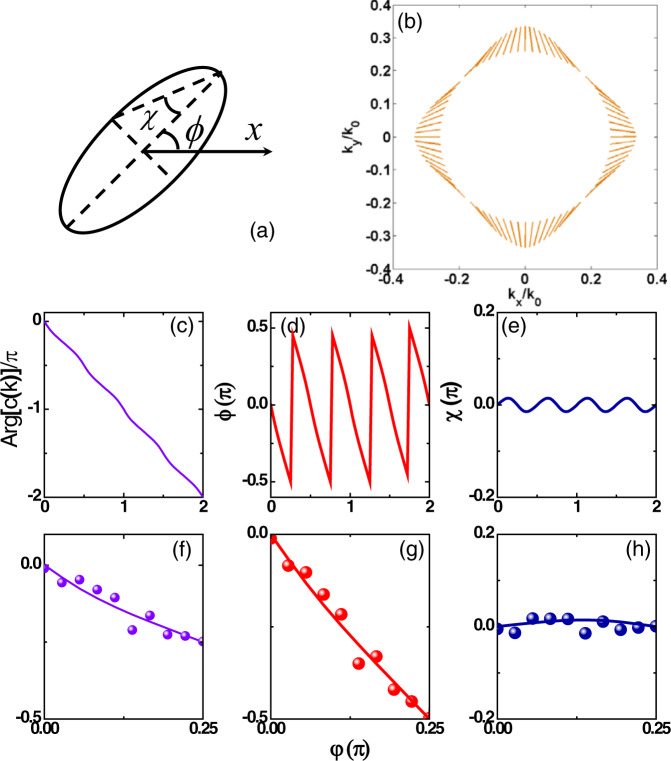
Polarization vortex of the BIC. **a** General schematic of the polarization ellipse *χ* and polarization angle *ϕ*. Direction of the polarization vector **c(k)** (**b**), angle of the polarization vector $arg[c({{{{{\bf{k}}}}}})]$ (**c**, **f**), polarization angle *ϕ* (**d**, **g**), and polarization ellipses *χ* (**e**, **h**) in momentum space for $\theta ={15}^{{{{{{\rm{o}}}}}}}$at the frequency of the 2nd BIC in the azimuthal direction. Solid lines illustrate the numerical results and the scatter points show the measured results.

The explored BIC supports a polarization vortex in the far field^48,51,59^. To characterize its topological character, we adopt the polarization vector ${{{{{\bf{c}}}}}}({{{{{\bf{k}}}}}})=\alpha ({{{{{\bf{k}}}}}}){e}^{i{\varphi }_{{{{{{\rm{TE}}}}}}}({{{{{\bf{k}}}}}})}{\hat{{{{{{\bf{e}}}}}}}}_{{{{{{\rm{TE}}}}}}}+\beta ({{{{{\bf{k}}}}}}){e}^{i{\varphi }_{{{{{{\rm{TM}}}}}}}({{{{{\bf{k}}}}}})}{\hat{{{{{{\bf{e}}}}}}}}_{{{{{{\rm{TM}}}}}}}$^48,51,59^, which refers to the polarization direction of this resonance in the far field, ${\varphi }_{{{{{{\rm{TE}}}}}},{{{{{\rm{TM}}}}}}}$ is the radiative phase to the TE (TM) polarization channel. Close to the BIC, under the assumption of a background response ${\chi }_{{{{{{\rm{TE}}}}}}}\approx {\chi }_{{{{{{\rm{TM}}}}}}}={\chi }_{0}$, ${\zeta }_{{{{{{\rm{TE}}}}}}}\approx {\zeta }_{{{{{{\rm{TM}}}}}}}={\zeta }_{0}$ at nearly-normal incidence, and lossless case ${\gamma }_{{{{{{\rm{d}}}}}}}=0$, ${{{{{\bf{c}}}}}}({{{{{\bf{k}}}}}})$ assumes the form ${e}^{i({\zeta }_{0}({{{{{\bf{k}}}}}})+{\chi }_{0}({{{{{\bf{k}}}}}})+\pi )/2}(\alpha ({{{{{\bf{k}}}}}}){\hat{{{{{{\bf{e}}}}}}}}_{{{{{{\rm{TE}}}}}}}+{e}^{{{{{{\rm{iN}}}}}}({{{{{\bf{k}}}}}})\pi }\beta ({{{{{\bf{k}}}}}}){\hat{{{{{{\bf{e}}}}}}}}_{{{{{{\rm{TM}}}}}}})$, which can be easily retrieved from the measured transmission coefficients assisted by $\beta {e}^{-iN\pi }/\alpha =-[({S}_{21}-{S}_{43})+\sqrt{{({S}_{21}-{S}_{43})}^{2}+4{S}_{23}{S}_{41}}]/(2{S}_{23})$ at the resonant frequency $\varDelta f=0$ [Eq. (2)] and performing a rotational transformation to bring it into the $({k}_{{{{{{\rm{x}}}}}}},{k}_{{{{{{\rm{y}}}}}}})$ space. For fixed $\theta =arc\,\sin [\sqrt{{k}_{{{{{{\rm{x}}}}}}}^{2}+{k}_{{{{{{\rm{y}}}}}}}^{2}}/{k}_{0}]$, the polarization vector direction $\arctan [{e}^{{{{{{\rm{iN}}}}}}({{{{{\bf{k}}}}}})\pi }\beta ({{{{{\bf{k}}}}}})/\alpha ({{{{{\bf{k}}}}}})]$ clearly shows a topological charge of the 2nd BIC is −1, as shown in Fig. 4b for $\theta ={15}^{{{{{{\rm{o}}}}}}}$. Here, ${k}_{0}=2\pi {f}_{{{{{{\rm{2ndBIC}}}}}}}/c$, ${f}_{{{{{{\rm{2ndBIC}}}}}}}$ is the frequency of the 2nd BIC and *c* is the light speed in vacuum. The angle $arg[c({{{{{\bf{k}}}}}})]$, shown in Fig. 4c, clearly shows that the phase variation is $-2\pi$ when the BIC is encircled, implying a topological charge of −1. The polarization angle *ϕ*, shown in Fig. 4d, indicates perfect polarization conversion between the two symmetric directions. The magnitude of the polarization ellipticity *χ*, as illustrated in Fig. 4e, is below 0.02, which implies almost linear polarization. The measured $arg[c({{{{{\bf{k}}}}}})]$, polarization angle *ϕ* and polarization ellipticity *χ* from the $\varGamma {{{{{\rm{X}}}}}}$ direction to the $\varGamma {{{{{\rm{M}}}}}}$ direction confirm this polarization distribution, as illustrated in Fig. 4f–h. The polarization vortex distribution with topological charge −1 indicates that this resonant mode has different radiative coupling between the two polarization channels along $\varGamma {{{{{\rm{X}}}}}}$ and $\varGamma {{{{{\rm{M}}}}}}$, which makes the critical coupling condition always satisfied at some point when the incident angle is continuously varied from the $\varGamma {{{{{\rm{X}}}}}}$ to the $\varGamma {{{{{\rm{M}}}}}}$ direction. In other words, due to the topological features of the BIC, we can always achieve CCPC at the critical coupling of resonance under symmetric incidence.

**Fig. 5:**
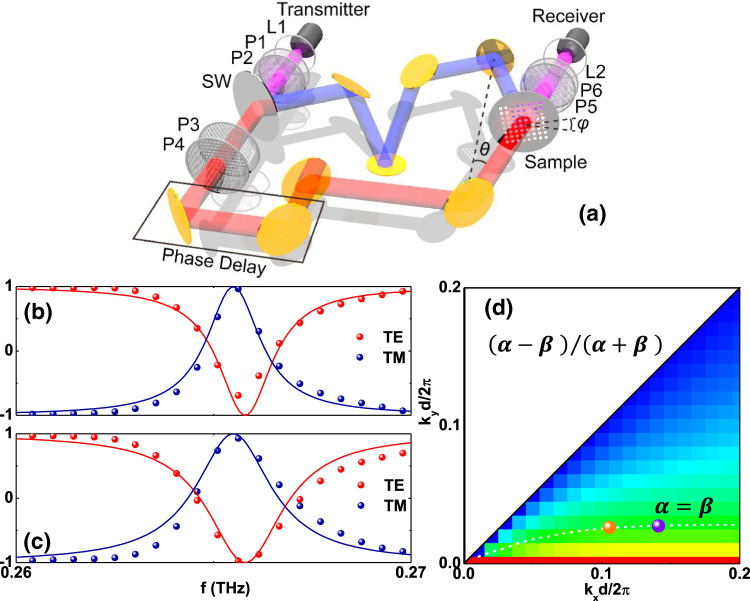
Coherent perfect polarization conversion. Experimental setup (**a**) and perfect polarization conversion under symmetric incidence at incident angles $(\theta ={15}^{{{{{{\rm{o}}}}}}},\varphi ={14}^{{{{{{\rm{o}}}}}}})$ (**b**) and $(\theta ={20}^{{{{{{\rm{o}}}}}}},\varphi ={11}^{{{{{{\rm{o}}}}}}})$ (**c**). The perfect polarization conversion point lies on the critical coupling curve in the contour plot (**d**), consistent with Fig. 2c. Fitted parameters are ${f}_{0}^{{{{{{\rm{TE}}}}}}}=0.2658$ THz, ${f}_{0}^{{{{{{\rm{TM}}}}}}}=0.2655$ THz, and ${\alpha }^{2}=0{.02027}^{2}$ THz for $(\theta ={15}^{{{{{{\rm{o}}}}}}},\varphi ={14}^{{{{{{\rm{o}}}}}}})$. Fitted parameters are ${f}_{0}^{{{{{{\rm{TE}}}}}}}=0.2658$ THz, ${f}_{0}^{{{{{{\rm{TM}}}}}}}=0.2655$ THz, and ${\alpha }^{2}=0{.024}^{2}$ THz for $(\theta ={20}^{{{{{{\rm{o}}}}}}},\varphi ={11}^{{{{{{\rm{o}}}}}}})$. *N* = 0 in all figures.

**Fig. 6:**
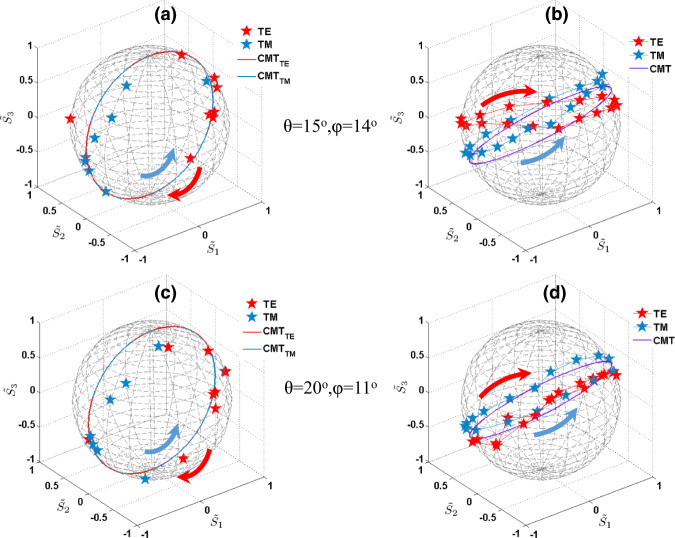
Experimental verification of polarization manipulation and its coherent control. Experimental results for coherent polarization state evolution when varying the frequency under symmetric TE/TM incidence with a fixed incident angle$(\theta ={15}^{{{{{{\rm{o}}}}}}},\varphi ={14}^{{{{{{\rm{o}}}}}}})$ (**a**) and $(\theta ={20}^{{{{{{\rm{o}}}}}}},\varphi ={11}^{{{{{{\rm{o}}}}}}})$ (**c**). Experimental coherent polarization state control when varying phase difference ψ under TE/TM incidence with a fixed incident angle $(\theta ={15}^{{{{{{\rm{o}}}}}}},\varphi ={14}^{{{{{{\rm{o}}}}}}})$ (**b**) and $(\theta ={20}^{{{{{{\rm{o}}}}}}},\varphi ={11}^{{{{{{\rm{o}}}}}}})$ (**d**) at resonant frequency $f=0.2656$ THz.

Next, we experimentally verify the generalized CPA condition to achieve arbitrary polarization conversion. Under symmetric TE/TM incidence, the resonance is excited when deviating from the symmetry-protected BIC. The symmetric TE/TM incidence means that the electric/magnetic field for the two incident beams is perpendicular to its corresponding plane of incidence, and oscillating in/anti phase with the same amplitude at the center plane of this symmetric slab. As we continuously change the incident angle between the $\varGamma {{{{{\rm{X}}}}}}$ and $\varGamma {{{{{\rm{M}}}}}}$ directions, perfect polarization conversion, can always be achieved. As sketched in Fig. 5a, a homemade fiber-based angle-resolved THz interferometer is adopted to experimentally measure the output light. At the incidence angle $\theta ={15}^{{{{{{\rm{o}}}}}}}$, by tuning the incidence azimuthal angle *φ* to $\varphi \approx {14}^{\circ }$ in the range from 0 to 45^o^, we find a narrow peak (dip) for ${\tilde{S}}_{{{{{{\rm{u}}}}}}1}$, as large (small) as 0.9632 (−0.6927) at $f=0.2656$ THz, which indicates perfect (near-perfect) polarization conversion under symmetric TM (TE) incidence ${\tilde{S}}_{{{{{{\rm{u}}}}}}1}=-1(1)$, as shown in Fig. 5b. The fitted CMT results agree well with the experimental results. The asymmetry for the two polarizations is possibly related to the non-radiative loss and a small asymmetry in the geometry of the fabricated slab (the radii of the holes on the top and bottom surfaces are around ${r}_{{{{{{\rm{t}}}}}}}=122.5$ μm and ${r}_{{{{{{\rm{b}}}}}}}=129$ μm, respectively), as discussed in Supplementary Notes 4 and 5. The condition for perfect polarization conversion in the Brillouin zone lies precisely on the critical coupling condition curve, as shown in Fig. 5d. For a different incident angle $\theta ={20}^{{{{{{\rm{o}}}}}}}$, we also find near-perfect polarization conversion for $\varphi \approx {11}^{\circ }$, with ${\tilde{S}}_{{{{{{\rm{u}}}}}}1}\approx 0.9294(-0.9708)$ around $f=0.2656$ THz for symmetric TM (TE) incidence, as illustrated in Fig. 5c, and this position also lies on the critical coupling curve in Fig. 5d. The corresponding output polarization states as a function of frequency for the symmetric incidence under the two selected incident angles, as illustrated in Fig. 6a, c, lie almost on the unit circle in the ${\tilde{S}}_{{{{{{\rm{u}}}}}}1}$–${\tilde{S}}_{{{{{{\rm{u}}}}}}3}$ plane, outlining the exciting opportunities for real-time polarization control. We should point out that coherent control requires momentum matching of the two incident beams. The impact of angular tolerance considering in-plane momentum mismatch under coherent control is discussed in Supplementary Note 6. To reduce the impact of momentum mismatch in the experimental setup, we carefully tuned the optical elements along the optical path. In addition, we stress that the angular response of our metasurface is broad, which implies that the performance of this device is not sensitive to small angular deviations generating momentum mismatch. Moreover, the measured polarization variation is also smooth, which confirms the insensitivity to momentum mismatch or unexpected variations in the control beams.

The output polarization state can also be dynamically manipulated by tuning the relative phase difference ψ, ranging from 0 to 2*π*, between the two incident beams. As an example, at the resonant frequency of the second guided mode $f={f}_{0}\approx 0.2656\;{{{{{\rm{THz}}}}}}$ for TE (TM) incidence at the angle $\theta ={15}^{{{{{{\rm{o}}}}}}},\,\varphi {=14}^{{{{{{\rm{o}}}}}}}$, the phase difference ψ drastically controls the polarization state, as shown in Fig. 6b. It must be emphasized that, although the input polarization state is fixed to a linear polarization point in the Poincare sphere, the output polarization state can be tuned over a closed curve from the input polarization state point. Similar polarization evolution can also be observed for other incident angles, e.g., $\theta ={20}^{{{{{{\rm{o}}}}}}},\,\varphi {=11}^{{{{{{\rm{o}}}}}}}$, in Fig. 6d. Consistent with the previous discussion, we note that the direct coupling does affect the output polarization state. Here, our fitted direct coupling is $|{r}_{0}|\approx 0.9336$ and *N* = 0, in good agreement with the experimental results shown in Fig. 6. Overall, we find that the output polarization state can be flexibly controlled by either changing the resonant frequency through geometrical changes in the design of our photonic crystal, as well as by tuning the phase difference between the two input waves under coherent control, which provides an excellent tool to dynamically manipulate the polarization state of the output beam.

## Discussion

To conclude, in this paper, we have introduced a generalized CPA condition for a freestanding silicon photonic crystal slab operated close to a symmetry-protected BIC. Its topological features ensure the opportunity to widely control the critical coupling condition with various knobs, and in turn, manipulate the polarization state of the output wave at will. Our work not only extends the concept of coherent polarization control to high Q functionalities, but also it demonstrates that polarization conversion around symmetry-protected BICs is not limited to linear polarizations, but through multiple wave excitations can be extended to arbitrary polarization control. Our findings are not limited to photonic crystal slabs, and they can be extended to other engineered systems supporting BICs, such as metasurfaces, guided wave structures, as well as acoustic and polaritonic systems.

## Methods

### Fabrication of the photonic crystal slab

Our freestanding photonic crystal slab is fabricated by the following processes: we generated a periodic hole pattern of photoresist on a 160 μm thick silicon slab by traditional lithography techniques, and we then etched the exposed silicon regions from top to bottom by a deep reactive ion etching approach, and finally removed the residual photoresist once the etching process is finished.

### Experimental setup

To measure the transmission under one port excitation, a fiber-based terahertz time-domain spectroscopy (TDS) system was applied (Advanced Photonics, Inc.), as displayed in Fig. 3a. The THz pulse generated by the photoconductive transmitter was firstly collimated by a TPX lens L1. After passing through the sample, it was collected by another TPX lens L2 and then focused onto the photoconductive receiver for detection. The two linear polarizers P1 and P2 (P3 and P4) before (after) the sample were used to tune (select) the incident (output) polarization to be either TE or TM polarized. The in-plane wave vector $({k}_{{{{{{\rm{x}}}}}}},{k}_{{{{{{\rm{y}}}}}}})$ of the incidence with respect to the sample was controlled by changing *θ* and *φ* using two rotators.

To measure the coherent output, a fiber-based terahertz TDS interferometer was built, as displayed in Fig. 5a. The THz pulse generated by the transmitter was first collimated by a TPX lens L1. It was then split into two beams (blue for the reflection beam and red for the transmission beam) by a high-resistance silicon wafer SW. After several gold mirror reflections, the two beams illuminated the sample with the same incident angle *θ* from opposite sides, where the sample was placed just at the beam-intersection position. The coherent output in one side was finally collected by another TPX lens L2 and focused onto the receiver for detection. To achieve desired incident in-plane wave vector $({k}_{{{{{{\rm{x}}}}}}},{k}_{{{{{{\rm{y}}}}}}})$, the in-plane rotation angle *φ* of the sample was further controlled using a rotation holder. The two linear polarizers P1 and P2 before the SW were used to control the incident polarization to be either TE or TM polarized. The two linear polarizers P3 and P4, in the transmission beam after the SW, were used to adjust the amplitude of this beam to match that of the reflected beam at the sample position, and meanwhile, maintain the incident polarization state. The two linear polarizers P5 and P6 between the sample and L2 were used to separately select the TE and TM polarization components of the coherent output beam for detection, which could be used to calculate the polarization state. To tune the relative phase delay between the two incident beams, a built-in delay line composed by two reflection mirrors on a translation stage was designed in the transmission beam.

Here, in our fiber-based THz system, the temporal scan was 320 ps, which almost covered the entire useful light signals. In order to increase the signal spectral resolution in the frequency range, we add an additional zero array in the next 1600 ps, where the original spectrum can be interpolated to give more frequency points.

### Reporting summary

Further information on research design is available in the Nature Research Reporting Summary linked to this article.

## Supplementary information

Supplementary Materials

Reporting Summary

## Data Availability

Data available upon request from the corresponding authors.
