# Supplementary material for: Coherent full polarization control based on bound states in the continuum
Source: Nat Commun. 2022 Aug 4;13:4536. doi: 10.1038/s41467-022-31726-1 (PMC9352794; doi:10.1038/s41467-022-31726-1)
Supplement: Supplementary file 1 — Supplementary Materials [file 41467_2022_31726_MOESM1_ESM.pdf]

Supplemental materials

Supplementary Note 1. The coupled-mode theory (CMT) description for the resonant guide mode

The optical features of resonant guided modes supported by a photonic crystal slab can be described using coupled-mode theory (CMT). As a starting point, we explore coherent complete polarization conversion (CCPC) enabled by these resonant guided modes using CMT, in which cross-polarization coupling is treated as a loss channel. We assume that the slab supports one resonant mode with complex amplitude q coupled to propagating plane waves sustaining radiation loss associated only with transverse-electric (TE) and transverse-magnetic (TM) zero-th order diffraction channels in the frequency range of interest, where the TE (TM) means that the electric (magnetic) field is perpendicular to the plane of incidence. The resonant response can be described in the steady-state regime by

$$\begin{aligned}[i\Delta f + \gamma]q &= \mathbf{K}\mathbf{a} \\ \mathbf{b} &= \mathbf{C}\mathbf{a} + \mathbf{D}^{\mathrm{T}} q\end{aligned}, \tag{S1}$$

where $\Delta f = f - f_0$ is the detuning from the resonance frequency f_0, and γ is the total decay rate, including all loss channels. The input and output waves are $\mathbf{a} = \begin{pmatrix} a_{\mathrm{u}}^{\mathrm{TE}} & a_{\mathrm{d}}^{\mathrm{TE}} & a_{\mathrm{u}}^{\mathrm{TM}} & a_{\mathrm{d}}^{\mathrm{TM}} \end{pmatrix}^{\mathrm{T}}$ and $\mathbf{b} = \begin{pmatrix} b_{\mathrm{u}}^{\mathrm{TE}} & b_{\mathrm{d}}^{\mathrm{TE}} & b_{\mathrm{u}}^{\mathrm{TM}} & b_{\mathrm{d}}^{\mathrm{TM}} \end{pmatrix}^{\mathrm{T}}$, where the superscripts TE and TM correspond to the input and output polarizations, and the subscripts u and d denote the waves in the upper and lower semi-space with respect to the slab, respectively. The matrix $\mathbf{C}$ describes the direct coupling between input and output waves when they do not interact with the resonant mode, *i.e.*, they interact with a non-resonant homogeneous isotropic slab without polarization conversion, so $\mathbf{C}$ is a matrix with

$$\mathbf{C} = \begin{pmatrix} \mathbf{C}^{\mathrm{TE}} & 0 \\ 0 & \mathbf{C}^{\mathrm{TM}} \end{pmatrix},\ \mathbf{C}^{\mathrm{TE}} = \begin{pmatrix} r_0^{\mathrm{TE}} & t_0^{\mathrm{TE}} \\ t_0^{\mathrm{TE}} & r_0^{\mathrm{TE}} \end{pmatrix},\ \mathbf{C}^{\mathrm{TM}} = \begin{pmatrix} r_0^{\mathrm{TM}} & t_0^{\mathrm{TM}} \\ t_0^{\mathrm{TM}} & r_0^{\mathrm{TM}} \end{pmatrix}. \tag{S2}$$

In the absence of material dissipation, energy conservation and reciprocity require that $\mathbf{C}^{\mathrm{TE,TM}}$ are unitary. We can assume that the transmission and reflection coefficients of the non-resonant slab are $r_0^{\mathrm{TE,TM}} = e^{i\zeta^{\mathrm{TE,TM}}}\cos(\chi^{\mathrm{TE,TM}})$, $t_0^{\mathrm{TE,TM}} = e^{i\left(\zeta^{\mathrm{TE,TM}}+\pi/2\right)}\sin(\chi^{\mathrm{TE,TM}})$, where $\zeta^{\mathrm{TE,TM}}$ are

global phase factors with respect to the reference plane, and $\chi^{\mathrm{TE,TM}}$ indicate the amplitude, with $\left|r_0^{\mathrm{TE,TM}}\right|^2+\left|t_0^{\mathrm{TE,TM}}\right|^2=1$. Mirror symmetry along the z-direction requires the resonant mode to be even or odd with respect to z, denoted by $\sigma = +1$ and -1, respectively. Due to energy conservation and time-reversal symmetry, the radiative coupling between the resonant mode and the input waves needs to be met additional requirements $\mathbf{K}=\mathbf{D}$ and $\mathbf{C}\mathbf{K}^{\dagger}+\mathbf{K}^{\mathrm{T}}=0$.

The radiative coupling is

$$\mathbf{K}=\left(\alpha e^{i\left(\zeta^{\mathrm{TE}}+\sigma\chi^{\mathrm{TE}}+\pi\right)/2},\ \sigma\alpha e^{i\left(\zeta^{\mathrm{TE}}+\sigma\chi^{\mathrm{TE}}+\pi\right)/2},\ \beta e^{i[\mathrm{N}\pi+\left(\zeta^{\mathrm{TM}}+\sigma\chi^{\mathrm{TM}}+\pi\right)/2]},\ \sigma\beta e^{i[\mathrm{N}\pi+\left(\zeta^{\mathrm{TM}}+\sigma\chi^{\mathrm{TM}}+\pi\right)/2]}\right), \tag{S3}$$

where α (β) is the radiative coupling coefficient for TE (TM) polarized waves and N is an integer. The scattering matrix $\mathbf{S}$, defined by $\mathbf{b}=\mathbf{S}\mathbf{a}$, can be expressed as

$$\mathbf{S}=\mathbf{C}+\frac{\mathbf{K}^{\mathrm{T}}\mathbf{K}}{i(\Delta f+\gamma)}=\begin{pmatrix}\mathbf{S}^{\mathrm{TE}} & \mathbf{S}^{\mathrm{C}}\\ \mathbf{S}^{\mathrm{C}} & \mathbf{S}^{\mathrm{TM}}\end{pmatrix}, \tag{S4}$$

where $\mathbf{S}^{\mathrm{TE}}$ ($\mathbf{S}^{\mathrm{TM}}$) indicates the scattering matrix of the resonant mode in the same polarization channel and $\mathbf{S}^{\mathrm{C}}$ indicates the cross-polarized scattering matrix. The total loss rate $\gamma=\alpha^2+\beta^2+\gamma_{\mathrm{d}}$, where γ_{d} is the dissipation loss rate.

Supplementary Note 2. Polarization conversion/absorption under one port excitation

The optical properties of this resonant mode has been described by Eq. S4. Under one port excitation, such as for the TE incidence, the effective loss in the same polarization channel is

$$\mathrm{A}_{\mathrm{TE}}=2\alpha^2\left(\beta^2+\gamma_{\mathrm{d}}\right)\Big/\left[\left(\Delta f\right)^2+\left(\alpha^2+\beta^2+\gamma_{\mathrm{d}}\right)^2\right]. \tag{S5}$$

This absorption has an upper bound of 0.5 in a mirror-symmetric resonator at critical coupling condition

$$\Delta f=0, \qquad \alpha^2=\beta^2+\gamma_{\mathrm{d}}. \tag{S6}$$

This upper bound makes it impossible to entirely absorb light or totally convert polarization for a single input wave due to symmetry. Take a polarization conversion as an example, the polarization conversion efficiency defined as the power in the crossed polarization for the transmitted or reflected wave with respect to the incident power has a theoretical upper limit

25% when there is no non-radiative loss. In order to overcome this bound and maximize absorption or polarization conversion, mirror symmetry can be broken, but this requires a trade-off with the overall footprint and fabrication complexity. Excitation with multiple waves can also overcome this bound and realize full absorption or polarization conversion, as shown in the case of coherent perfect absorptions (CPAs). Coherent control has indeed emerged as an attractive approach to enable unitary absorption in ultrathin resonant systems, also offering the opportunity of dynamic control by tailoring the relative phase between the inputs.

Supplementary Note 3. The Q factors retrieved from the experimental transmission data through the CMT fitting

To character the experimental resonance in detail, we extract the Q factor of the explored resonance. We fit experimentally measured transmitted spectra in Fig. 3 with the expression obtained from Eq. S4. For the explored resonance, the fitted transmitted intensity formulas are in general

$$T_{\mathrm{TE}}=|t_{\mathrm{TE}}|^2=|S_{21}|^2=\left|\frac{-\alpha^2 e^{i\chi^{\mathrm{TE}}}}{i\Delta f+\alpha^2+\beta^2+\gamma_{\mathrm{d}}}+i\mathrm{Sin}\left[\chi^{\mathrm{TE}}\right]\right|^2,$$

$$T_{\mathrm{TM}}=|t_{\mathrm{TM}}|^2=|S_{43}|^2=\left|\frac{-\beta^2 e^{i\chi^{\mathrm{TM}}}}{i\Delta f+\alpha^2+\beta^2+\gamma_{\mathrm{d}}}+i\mathrm{Sin}\left[\chi^{\mathrm{TM}}\right]\right|^2,$$

$$T_{\mathrm{C}}=|t_{\mathrm{C}}|^2=|S_{41}|^2=\left|\frac{\alpha\beta}{i\Delta f+\alpha^2+\beta^2+\gamma_{\mathrm{d}}}\right|^2. \tag{S7}$$

The radiative quality factor is $\mathrm{Q_r}=f_0\big/\left[2\alpha^2\left(\beta^2\right)\right]$ in the original excitation TE (TM) polarization channel, and the corresponding non-radiative Q factor is $\mathrm{Q_{nr}}=f_0\big/\left[2\gamma_{\mathrm{d}}+2\beta^2\left(\alpha^2\right)\right]$. Along the $\Gamma\mathrm{X}$ direction, we can only use T_{TE} to fit experimentally measured transmission around the explored resonance under TE incidence due to $\beta=0$. Along the $\Gamma\mathrm{M}$ direction, we can only use T_{TM} to fit experimentally measured transmission around the same resonance under TM incidence due to $\alpha=0$. $\mathrm{Sin}\left[\chi^{\mathrm{TE,TM}}\right]$ is the transmission amplitude of a homogeneous background slab whose permittivity can be approximately equal to the average of the PhC slab. By fitting the transmitted intensity at each

incident direction, we obtain the radiative Q factor $\mathrm{Q_r}$ and non-radiative Q factor $\mathrm{Q_{nr}}$ both along $\Gamma\mathrm{X}$ and $\Gamma\mathrm{M}$ directions, as shown in Fig. 3(d). In Fig. S1, we provide the fitting and measured transmitted intensities at $\theta = 15^{\mathrm{o}}$ as a concrete example. Around this explored resonance, the fitting results agree with the measured results.

Supplementary Note 4. Effects of the nor-radiative loss

In the ideal zero non-radiative loss rate, we have pointed that the perfect polarization conversion phenomenon with 100% efficiency coincides with the pseudo CPA phenomenon under symmetric incidence. However, the non-radiative loss cannot be fully ignored in practice due to the dissipation loss from material absorption, scattering from imperfect fabrication, finite excitation area of the slab and other interfering factors. The effect of finite non-radiative loss rate, $\gamma_{\mathrm{d}} \neq 0$, firstly leads to the critical coupling condition splitting in the parameter space for the excited TE and TM incidences, $\alpha^2 = \beta^2 + \gamma_{\mathrm{d}}$ for the TE incidence while $\beta^2 = \alpha^2 + \gamma_{\mathrm{d}}$ for the TM incidence. This also means the CCPC position occurs at different position in the parameter space for the TE and TM incidences. When the non-radiative rate is small with respect to the radiative loss rate, $\gamma_{\mathrm{d}} \ll \beta^2\left(\alpha^2\right)$, this splitting is not obvious in the parameter space. Besides the critical coupling condition splitting, the non-radiative loss rate does not break the presence of the CCPC but will decrease the power in the crossed polarization channel, *i.e.* decrease the conversion efficiency of the complete polarization conversion. For example, the conversion efficiency defined by the output power in the crossed polarization channel with respect to the incident power in each side can be evaluated by $\beta^2 \big/ \left(\beta^2 + \gamma_{\mathrm{d}}\right)$ at the critical coupling condition for the TE incidence, while the efficiency of the non-radiative contribution is $\gamma_{\mathrm{d}} \big/ \left(\beta^2 + \gamma_{\mathrm{d}}\right)$. To increase the conversion efficiency of the CCPC, we need to decrease the non-radiative loss rate.

To explore the effect of the non-radiative loss rate in our fabricated slab around the CCPC, we need to retrieve both the radiative and non-radiative Q factors around each CCPC. At the first measured CCPC position (θ=15°, $\varphi = 14^{\circ}$), the measured transmission and fitting spectra

including the crossed polarization comment are displayed in Figs. S2(a)-(c) with these fitting parameters: f_0=0.2658, α^2=0.02027^2, $\beta^2 = 0.0183^2$, and $\gamma_{\rm d} = 0.000077$ in units of THz. Due to $\gamma_{\rm d} \ll \beta^2\left(\alpha^2\right)$, we expect the splitting of the critical condition is small. And $\alpha \approx \beta$ with almost zero $\gamma_{\rm d}$, these microscopic parameters at this position almost satisfy the critical coupling condition, which implies near zero output power in the original excited polarization channel, as shown in Figs. S2(d)-(f). Owing to the finite value of $\gamma_{\rm d}$, the conversion efficiency of this CCPC cannot be perfect in the amount of $\eta = \beta^2/\alpha^2 = 81.26\%$, which agrees with the measured efficiency 71.15%. The sight difference may be due to the fact that the measured frequency point is not exactly on the resonance frequency with small deviation Δf = -0.0002 THz and some other reasons.

For the second CCPC position we measured, ($\theta = 20^{\rm o}, \varphi = 11^{\rm o}$), these fitting parameters are: $f_0 = 0.2658$, $\beta^2 = 0.0223^2, \alpha^2 = 0.024^2$, and $\gamma_{\rm d} = 0.000077$ in units of THz by fitting the measured transmission spectra through the CMT, as shown in Figs. S3(a)-(c). We also expect the splitting of the critical condition is small due to $\gamma_{\rm d} \ll \beta^2\left(\alpha^2\right)$, and these retrieved microscopic parameters at this position almost satisfy the critical coupling condition, which is experimentally confirmed by near zero output power in the original excited polarization channel under symmetric incidence, as shown in Figs. S3(d)-(f). The theoretical conversion efficiency $\eta = \beta^2/\alpha^2 = 86.63\%$ agrees well with the measured efficiency η=81.18%. We should point out that the measured conversion efficiency under one port incidence is 19.55% (20.06%) for the first (second) CCPC position, below the theoretical limit 25% even at the critical coupling condition. If the conversion efficiency defined by the output power in the crossed polarization in one side with respect to the total power both in the two sides, the measured conversion efficiency is about 35.58% (40.59%) for the first (second) CCPC position in our coherent approach, which shows obvious advantage to go beyond theoretical limit under one port excitation.

Supplementary Note 5. Effects of the imperfect fabrication

Our previous analysis assumes that the radius of the hole in the crystal slab is constant $r = 125.6$ μm along the z direction. In the fabricated slab, the radius of the holes on the top and bottom surfaces is around $r_t = 122.5$ μm and $r_b = 129$ μm, respectively. There exists slight deviation from our perfect slab assumption. We discuss the influence on the device performance due to this small variation. This small variation due the radius of the hole makes the slab deviating from the perfect mirror symmetry along the z direction. This imperfect z -mirror symmetry does not break the existence of the BIC at Γ point as the C_4 symmetry still be retained. This has been discussed in the Ref. [5], and our numerical simulations also clearly show the existence of our explored BIC, as illustrated in Fig. S4. But this imperfect z -mirror symmetry will lead to slightly different radiative coupling rates for the up and down sides when deviating from the BIC point. This further gives rise to slight difference for the reflection coefficients for the up and down sides, which leads to the zero output in the original polarization channel does not coincides with the symmetric incidence. We perform detailed full numerical simulations for the asymmetric hole case, the reflection on both sides around each CCPC point are plotted in Figs. S5(a) and (b). The difference is very small even at the resonance under such a hole radius variation. The output intensity for the down (up) side under symmetric TE incidence on each CCPC is still almost zero, 0.00516 (0.00105) around 0.2656 THz in Fig. S5(c) and 0.0014 (0.00249) around 0.2656 THz in Fig. S5(d). The effect of this small variation due the radius of the hole in our slab can be ignored.

Supplementary Note 6. Angular bandwidth

The coherent approach requires the two incident lights to match their in-plane momenta to achieve the desired polarization control. Once the in-plane momentum math condition is deviated, *i.e.* two different incident angles for the two incident lights, the deviated in-plane momentum match will affect the interference between the reflected and transmitted lights. To briefly discuss this impact, we adopt the angular spectra both in the θ and φ directions. Take the first measured CCPC point (θ=15°, φ=14°) at the resonance as an example, the

transmitted power in the crossed polarization channel along the θ (φ) direction with a fixed φ (θ) can be used to approximately evaluate the angular tolerance. As shown in Figs. S6(a) and (b), we find the angular spectra are wide both for the φ direction with θ=15° and the θ direction with $\varphi = 14^{\text{o}}$. The experimental measured results agree with the numerical results in terms of the profile. The peak value of the measured result is lower than the numerical result is due to the fact the non-radiative loss is not included in our numerical simulation. Our numerical and experimental results indicate that the angular bandwidth with 90% is above 3°, we believe this can be easily satisfied in the experimental setup. In addition, we report measured polarization states for the transmission under TE incidence, as displayed in Fig. S7. The polarization does not show prominent variations with respect to small changes in the incident angle around the first CCPC point. We also find that perfect polarization conversion does not occur in this symmetric device under one port incidence case, which also implies that the range of polarization manipulation can be expanded under coherent control.

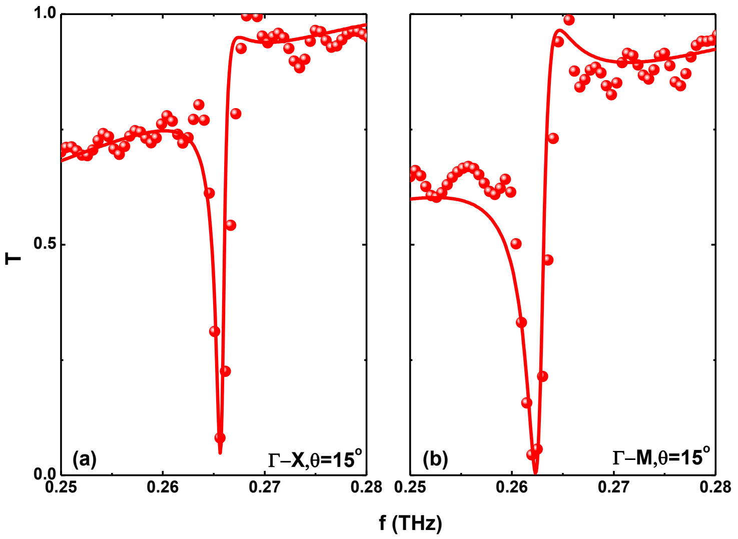

Figure S1. Measured (Scatter) and CMT fitting (Solid line) transmitted intensities along the ΓX (a) and ΓM (b) directions when the incident angle is $\theta = 15^{\circ}$. In (a), $f_0 = 0.2658$, $\alpha^2 = 0.0192^2$, $\beta^2 = 0$ and $\gamma_{\mathrm{d}} = 0.000121$ in units of THz. In (b), $f_0 = 0.2628$, $\alpha^2 = 0$, $\beta^2 = 0.029^2$ and $\gamma_{\mathrm{d}} = 0.000077$ in units of THz.

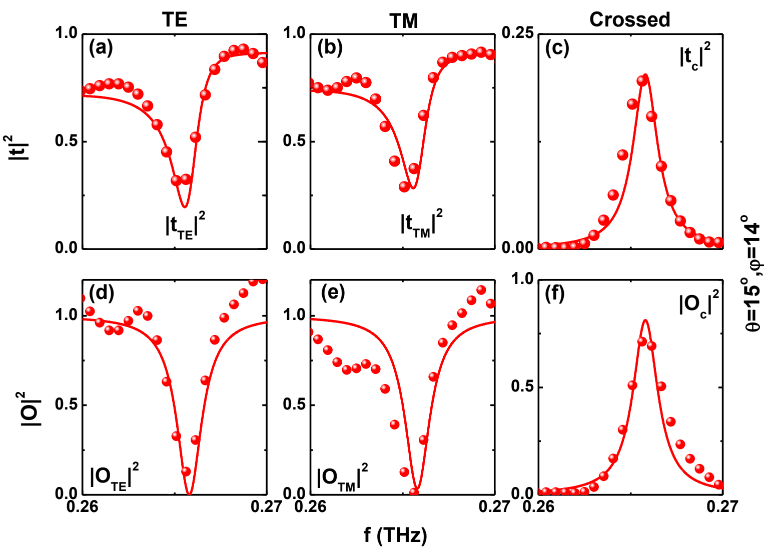

Figure S2. The experimental and CMT fitting transmitted intensities in the original excited TE(a) and TM (b) incident polarization channel and the crossed polarization channel (c) around the 1st CCPC position $(\theta = 15^{\circ}, \varphi = 14^{\circ})$. The experimental and CMT fitting output intensities in the original excited TE (c) and TM (d) incident polarization channel and the crossed polarization channel (e) around the 1st CCPC position $(\theta = 15^{\circ}, \varphi = 14^{\circ})$ under symmetric incidence.

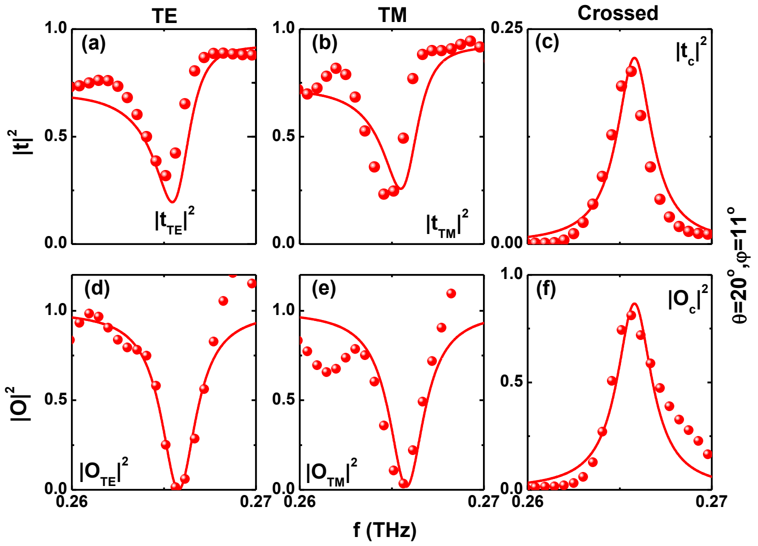

Figure S3. The experimental and CMT fitting transmitted intensities in the original excited TE(a) and TM (b) incident polarization channel and the crossed polarization channel(c) around the 2nd measured CCPC position $(\theta = 20^{\circ}, \varphi = 11^{\circ})$. The experimental and CMT fitting output intensities in the original excited TE(c) and TM (d) incident polarization channel and the crossed polarization channel (e) around the 2nd CCPC position $(\theta = 20^{\circ}, \varphi = 11^{\circ})$ under symmetric incidence.

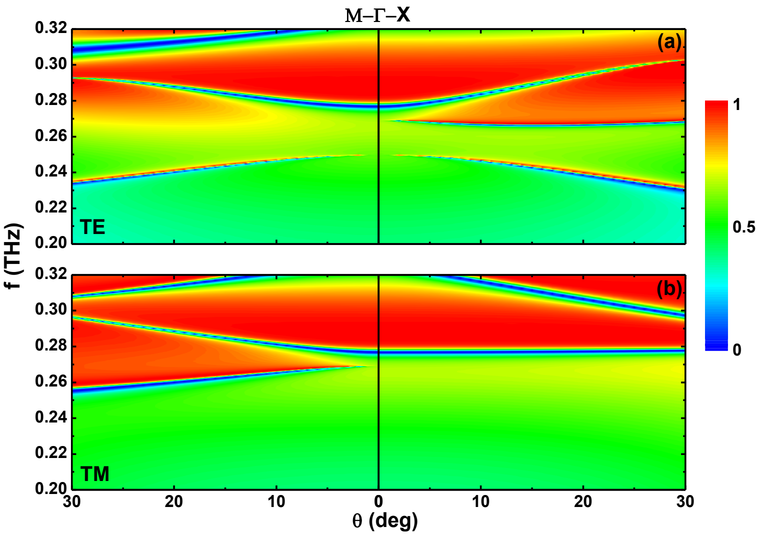

Figure S4. Numerical transmission spectra along the symmetry directions for incident TE (a) and TM (b) polarized light in a photonic crystal slab with slightly asymmetric hole.

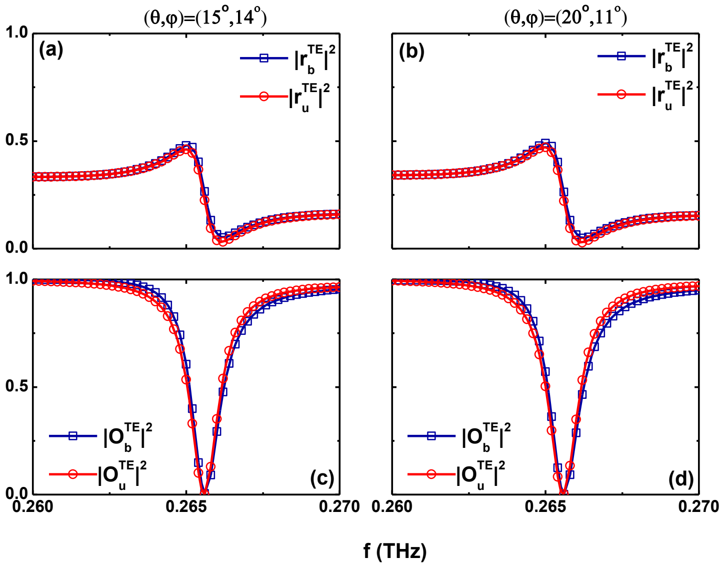

Figure S5. The reflection intensities for the top and bottom sides around the 1st CCPC position (a) and the 2nd CCPC (b) position in the asymmetric hole slab. The output intensities for the up and down sides in a photonic crystal slab with slightly asymmetric hole around the first CCPC position (a) and the 2nd CCPC (b) position under the symmetric TE incidence.

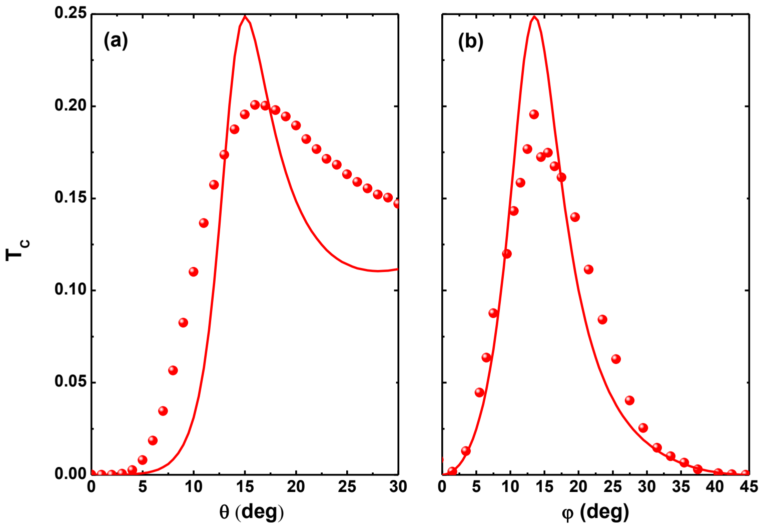

Figure S6. Experimental (Scatter) and numerical (Solid) transmitted intensities in the crossed polarization channel under TE incidence along the θ direction with a fixed $\varphi = 14^{\circ}$ (a) and along the φ direction with a fixed $\theta = 15^{\circ}$ (b).

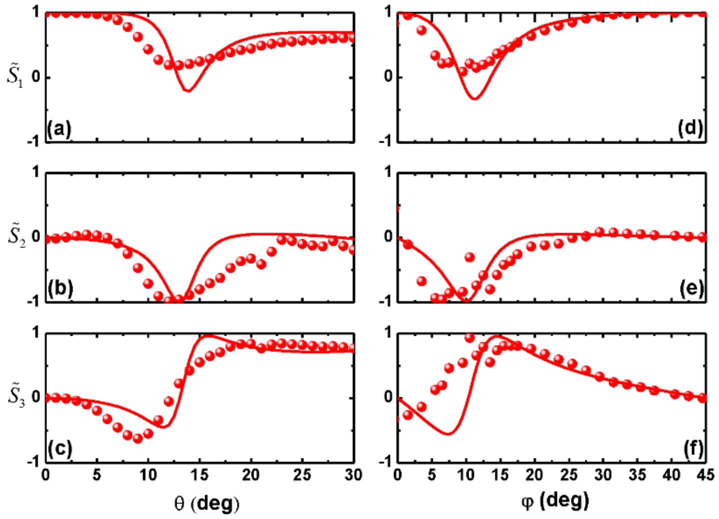

Figure S7. Experimental (Scatter) and numerical (Solid) transmitted normalized Stokes parameters under TE incidence along the θ direction with a fixed $\varphi = 14^{\circ}$ (a)-(c) and along the φ direction with a fixed $\theta = 15^{\circ}$ (d)-(f).
